# Two hits in one: whole genome sequencing unveils LIG4 syndrome and urofacial syndrome in a case report of a child with complex phenotype

**DOI:** 10.1186/s12881-016-0346-7

**Published:** 2016-11-17

**Authors:** Abeer Fadda, Fiza Butt, Sara Tomei, Sara Deola, Bernice Lo, Amal Robay, Alya Al-Shakaki, Noor Al-Hajri, Ronald Crystal, Marios Kambouris, Ena Wang, Francesco M. Marincola, Khalid A. Fakhro, Chiara Cugno

**Affiliations:** 1 Biomedical Informatics Division, Research Branch, Sidra Medical and Research Center, Doha, Qatar; 2 Hamad Medical Corporation, Doha, Qatar; 3 Division of Translational Medicine, Research Branch, Sidra Medical and Research Center, Doha, Qatar; 4 Weill Cornell Medicine in Qatar, Doha, Qatar; 5 Weill Cornell Medicine, New York, NY USA; 6 Division of Genetics, Department of Pathology, Sidra Medical and Research Center, Doha, Qatar; 7 Department of Genetics, Yale University School of Medicine, New Haven, CT USA; 8 SIDRA Medical and Research Center, Clinical Research Center, Out-Patient Clinic, Al Luqta Street, Education City, North Campus Qatar Foundation, PO Box 26999, Doha, Qatar

**Keywords:** LIG4, LRIG2, Immunodeficiency, Microcephaly, Vesicoureteral reflux, Whole genome sequencing, Case report

## Abstract

**Background:**

Ligase IV syndrome, a hereditary disease associated with compromised DNA damage response mechanisms, and Urofacial syndrome, caused by an impairment of neural cell signaling, are both rare genetic disorders, whose reports in literature are limited. We describe the first case combining both disorders in a specific phenotype.

**Case presentation:**

We report a case of a 7-year old girl presenting with a complex phenotype characterized by multiple congenital abnormalities and dysmorphic features, microcephaly, short stature, combined immunodeficiency and severe vesicoureteral reflux. Whole Genome Sequencing was performed and a novel ligase IV homozygous missense c.T1312C/p.Y438H mutation was detected, and is believed to be responsible for most of the clinical features of the child, except vesicoureteral reflux which has not been previously described for ligase IV deficiency. However, we observed a second rare damaging (nonsense) homozygous mutation (c.C2125T/p.R709X) in the leucine-rich repeats and immunoglobulin-like domains 2 gene that encodes a protein implicated in neural cell signaling and oncogenesis. Interestingly, this mutation has recently been reported as pathogenic and causing urofacial syndrome, typically displaying vesicoureteral reflux. Thus, this second mutation completes the missing genetic explanation for this intriguing clinical puzzle. We verified that both mutations fit an autosomal recessive inheritance model due to extensive consanguinity.

**Conclusions:**

We successfully identified a novel ligase IV mutation, causing ligase IV syndrome, and an additional rare leucine-rich repeats and immunoglobulin-like domains 2 gene nonsense mutation, in the context of multiple autosomal recessive conditions due to extensive consanguinity. This work demonstrates the utility of Whole Genome Sequencing data in clinical diagnosis in such cases where the combination of multiple rare phenotypes results in very intricate clinical pictures. It also reports a novel causative mutation and a clinical phenotype, which will help in better defining the essential features of both ligase IV and leucine-rich repeats and immunoglobulin-like domains 2 deficiency syndromes.

**Electronic supplementary material:**

The online version of this article (doi:10.1186/s12881-016-0346-7) contains supplementary material, which is available to authorized users.

## Background

DNA ligase IV (LIG4, OMIM#601837) is located on chromosome 13.q33-q34 and encodes a polypeptide of 911 amino acids [1]. It is essential for embryonic development and its complete deficiency causes early lethality accompanied by defective lymphogenesis and defective neurogenesis in knock-out mice [2, 3].

LIG4 is a component of the nonhomologous end-joining (NHEJ) complex which is the major DNA double-strand break (DSB) repair mechanism in human cells in response to ionizing radiation as well as in V(D)J recombination [4]. Along with other genes, such as Artemis, X-Ray Repair Cross-Complementing Protein 4 (XRCC4), Protein Kinase DNA activated Catalytic Polypeptide (PRKDC), and NHEJ1, encoding V(D)J/NHEJ proteins, it has been found to be deficient in human genetic disorders.

Ligase IV (LIG4) syndrome (OMIM #606593) is an autosomal recessive disorder of DNA damage repair, with cellular hypersensitivity to ionizing radiation, caused by mutations in LIG4. The disease is highly heterogeneous in terms of clinical presentation, i.e., various degrees of immunodeficiency, ranging from no immunodeficiency to profound Severe Combined Immunodeficiency (SCID) phenotypes, have been reported so far. Riballo et al. described the first patient with a defect in NHEJ in 1999 [5]. This Turkish patient, without overt immunodeficiency, developed acute T cell lymphoblastic leukemia at age 14 and was recognized as LIG4 deficient because of an over-response to radiotherapy. After this first leukemia patient, several other patients have been identified with LIG4 deficiency and lymphoproliferation or lymphoid malignancies: EBV associated B cell lymphoma in two patients [6, 7] and acute T cell leukemia in another patient [8]. Two additional series of patients have been shown to exhibit developmental delay and microcephaly along with mild degrees of immunodeficiency [6, 8–10] and SCID, respectively [7, 11–13]. Recently Murray et al. [14] updated these published 15 cases with an additional report of 10 LIG4 deficient cases from 9 families all sharing a similar phenotype (extreme growth retardation, microcephaly and mild immunodeficiency) and genotype (recurrence of p.R278H mutation in heterozygosity with a second variable mutation).

LIG4 mutations have been also identified in 2 patients diagnosed with Dubowitz syndrome (OMIM # 223370), suggesting that defective DNA repair triggers the development of at least a subset of this syndrome, offering a molecular basis for this poorly understood hereditary disease [15]. Dubowitz syndrome is an autosomal recessive disorder with a unique set of clinical features, including microcephaly, growth failure, mild mental retardation, eczema, immunodeficiency, radiosensitivity with an increased risk of blood dyscrasia and malignancy, and distinct facial features, which clearly overlap with the phenotypes of other genetic diseases of defective DNA repair, including LIG4 syndrome [16].

Leucine-rich repeats and immunoglobulin-like domains 2 gene (LRIG2, OMIM#608869) is located on chromosome 1p13.2 and encodes a protein of 1065 amino acids implicated in neural cell signaling and oncogenesis. Recently, Stuart et al. [17] discovered mutations in LRIG2 to be the cause of a subset of Urofacial syndromes (UFS2; OMIM#615112), an autosomal recessive disorder characterized by congenital urinary bladder dysfunction along with an abnormal facial expression upon smiling, laughing, and crying. Affected individuals usually experience lifelong urinary incontinence, recurrent urosepsis, vesicoureteral reflux (VUR), and renal failure. Some UFS2 patients also have severe constipation, indicating a generalized elimination defect [17, 18].

## Case presentation

### Clinical report

**Fig. 1:**
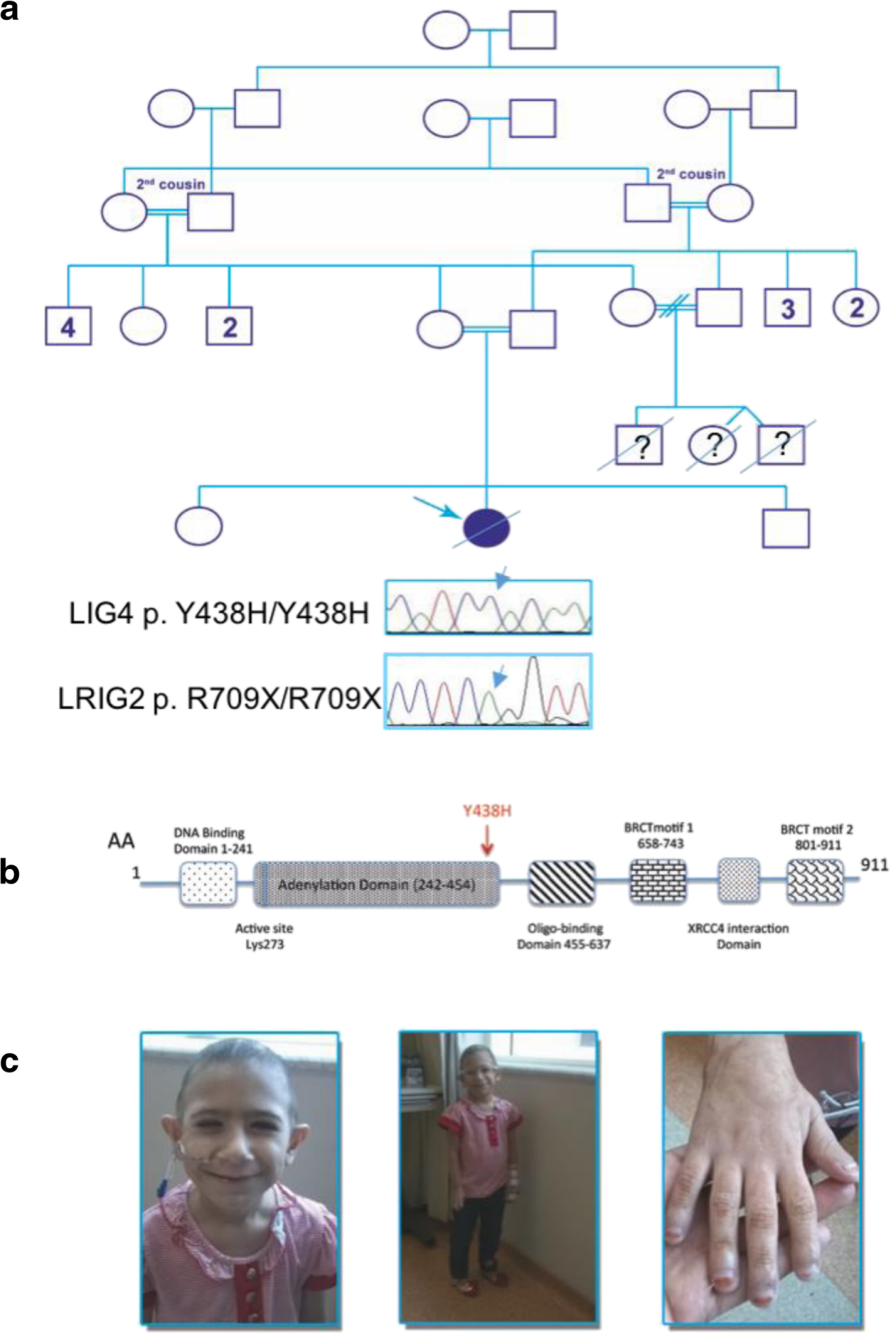
**a**. Family pedigree: the proband is highlighted by the *arrow*; LIG4 and LRIG2 chromatograms are shown for the homozygous proband; (**b**). LIG4 protein domains: the Y438H mutation is located in the adenylation domain (modified from Chistiakov [21]); (**c**). Pictures of the patient showing generalized hyposomia, facial dysmorphic features, but not inverted smile, and nail dystrophy

A Syrian 7-year old girl was referred to us for suspected immunodeficiency of unknown etiology in a clinical context of multiple congenital abnormalities and developmental delay. She belonged to a family of Palestinian ethnicity, characterized by a high degree of consanguinity in two consecutive generations; in particular, the parents were first cousins. An 8-year old sister and a 3-year old brother were healthy; three first cousins, also born from a consanguineous marriage, died of recurrent, severe infections before age 2, lacking any diagnosis in Syria (Fig. 1a).

The girl was born by normal vaginal delivery, after a spontaneous and uneventful pregnancy; she was full term and small for gestational age (birth body weight (BW) 2.5 kg, <3^rd^ percentile at the 39^th^ week of gestation). She required early admission to NICU, on day 2, for respiratory distress secondary to infective pneumonia and feeding problems with poor suckling. Thereafter, she experienced recurrent infections: lower respiratory infections with wheezing approximately every 3–4 weeks, suppurative otitis media, sinusitis, and oral candidiasis. At least three bacterial pneumonia episodes have been radiologically documented from 2013 to 2014, during the permanence of the child in Qatar, and, as a result of the recurrence of the respiratory infections, the chest MRI performed in February 2014, showed left lower lobe collapse and consolidation and chronic mild pleural effusions. No anatomical abnormalities, potentially predisposing recurrent lower respiratory tract infections were detected. During the previously mentioned time lapse, chronic sinusitis and recurrent suppurative otitis media were diagnosed, and *Streptococcus pneumoniae* and *Haemophilus influenzae* were isolated in several ear swabs. The brain MRI in February 2014 detected bilateral otomastoiditis and pansinusitis. Besides the infectious complications that required broad spectrum antibiotic treatment, the patient was presenting feeding difficulties (poor oral food intake and failure to thrive), requiring nutritional support with enteral feeding through a nasal gastric tube.

Starting from the age of 4, the child developed 4 episodes of urosepsis, secondary to severe vesicoureteral reflux (grade IV-V) and leading to left kidney hypoplasia and scarring, as documented in the urethrocystography in November 2013 and abdomen MRI in April 2014. She also presented with primary nocturnal enuresis but not incontinence.

Physical examination showed generalized hyposomia (BW and stature <3^rd^ percentile for age) and microcephaly (head circumference < <3^rd^ percentile for age). Accordingly, the radiological bone age was significantly delayed (3 ½ years at age of 6). The facial features included triangular face, small and slightly down-slanting eyes, hypotelorism, prominent nose with wide nasal pyramid, low-set and large ears and wide mouth. Moreover, she presented with sparse, thin hairs and nail dystrophy (Fig. 1c).

The skeletal survey revealed the presence of eleven pairs of ribs and bilateral finger/toe clubbing.

The patient had mild developmental delay with learning difficulties, underdeveloped language skills, and cognitive impairment, partly due to lack of schooling for the frequent hospitalizations. There was no history of developmental regression. Brain MRI showed normal parenchyma and ventricular system.

As for the lab tests, serial full blood counts from 2013 to 2014 pointed out a condition of persistent neutropenia (ranging from 500/mm^3^ to 1000/mm^3^), with a physiologic neutrophil response to acute infections (up to 2000/mm^3^), anemia (Hb ranging from 9 to 10 g/dl) and mild thrombocytopenia (PLT count between 90,000 and 130,000/mm^3^). Pancytopenia was revealed for the first time at age of 3, due to the appearance of bruises and petechial rash (PLT 50,000/mm^3^, Hb 7 g/dl, leucopenia not otherwise specified).

The level of immunoglobulins (Ig) and Ig subclasses surprisingly revealed only mild hypo-IgG2 and -IgM (IgG 1050 mg/dl (n.v. 633–1280), IgA 353 mg/dl (n.v. 33–202), IgM 22 mg/dl (n.v. 48–207), IgG1 954 mg/dl (n.v. 377–1130), IgG2 43.3 mg/dl (n.v. 68–388), IgG3 80.1 mg/dl (n.v. 15–89), IgG4 1.2 mg/dl (n.v. 1.2-169). Nevertheless, the flow cytometry count of T- and B-lymphocytes and NK cells, showed a severe B and T helper lymphopenia, as detailed in Table 1. The patient had an incomplete but severe block in precursor B cell differentiation, resulting in extremely low levels of blood B cells, whereas the less pronounced reduction of T cells was mainly caused by an increase in the CD8+ T cells, probably related to chronic infections. These findings were confirmed in a second test, after recovery from acute infections; no systemic steroids were being administered at that time.

**Table 1:** Patient’s immunophenotype assessed by flow cytometry: counts of B-lymphocytes, T lymphocytes with CD4+ helper and CD8+ cytotoxic subsets, and NK cells

T/B Lymphocytes and NK populations				
	cells/μL	n.v. cells/μL	%	n.v. %
CD3+	816 cells/μL	700–4200	89.0	55–78
CD4+	112 cells/μL	500–2000	10.9	27–53
CD8+	416 cells/μL	300–1800	40.8	19–34
CD19+	3 cells/μL	200–1600	0.3	10–31
CD56+	66 cells/μL	90–900	8.1	4–26
CD3 + CD4-CD8-			37.3	
CD4/CD8 ratio	0.26			

Normal karyotype, 46,XX was detected along with normal plasma amino acids screen and oxidative burst in both monocytes and neutrophils in response to stimulants, such as phorbol myristate acetate.

In early 2015, the patient left Qatar back to Syria, where she died of fatal bilateral pneumonia, and her immunological work-up remained incomplete, i.e. lymphocyte T-mitogen stimulation and the response to vaccinations could not be tested.

## Materials and methods

The venous blood samples were collected after informed consent and upon approval by the Joint IRB, WCM-Q & HMC, Doha, Qatar, protocol n. 13–00065, from six members of the family: namely, the affected girl, the two unaffected siblings, the mother and the maternal uncle and grandmother (father’s sample was not available at that time).

The DNA extracted from the patient and the family members was analyzed by Whole Genome Next Generation Sequencing [Illumina Hiseq 2500, 125 bp paired-end reads, 30× average coverage]. Sequence alignment and variant calling were performed utilizing the Illumina proprietary pipeline. Ingenuity® Variant Analysis software was utilized to filter low quality (<20), low coverage (<20) variants and those with allele frequency > 0.001 in 1000Genomes and ESP. Additional file 1: Table S1 describes the variants across the sequential filtering steps. The pedigree of this family suggested an autosomal recessive disease and, therefore, the data were mined for homozygous damaging variants, conforming to autosomal recessive inheritance. All reported variants were validated by Sanger sequencing.

Prediction of functional effects of mutations was done by SIFT (http://sift.jcvi.org) and PolyPhen-2 (http://genetics.bwh.harvard.edu/pph2/), the two tools employed by Ingenuity.

### Genetic analysis

Since a conclusive diagnosis was not reached based on the clinical presentation, we performed Whole Genome Sequencing (WGS). A novel LIG4 homozygous missense c.T1312C/p.Y438H mutation was detected and predicted to be damaging due to a tyrosine to histidine substitution in the adenylation domain of the enzyme. This domain binds ATP and contains many of the active-site residues (Fig. 1b). The DNA residue (c.T1312) was found to be conserved by the PhyloP program which compares genomes of 45 vertebrate species, with a *p*-value of 1.6 × 10^−5^.

Patient’s mother and siblings were healthy and heterozygous for this mutation.

We also found a pathogenic mutation in LRIG2, a homozygous nonsense substitution c.C2125T resulting in an arg709-to-ter (R709X) substitution, already reported to cause UFS [17].

Both autosomal recessive variants were present in a homozygous state in the patient and heterozygous state or absent in the tested family members. They were absent from all public databases including 1000 Genome Project, HapMap, Genome India and Genome Iceland and in 400 ethnically matched control chromosomes.

Due to the sudden departure of the patient prior to the disclosure of these results, we were not able to perform further functional experiments, such as assessing protein expression level of the mutant LIG4 enzyme, testing patient’s in vitro sensitivity to radiotherapy, etc.

## Conclusions

In our study, WGS unveiled the presence of multiple autosomal recessive conditions due to extensive consanguinity, in a young patient presenting a complex phenotype characterized by microcephaly, growth failure, immunodeficiency, dysmorphic features, skeletal abnormalities, cognitive delay, feeding difficulties and severe VUR, arising from the co-existence of two rare genetic diseases, LIG4 syndrome and UFS.

Our patient carried a previously unknown homozygous mutation (c.T1312C/p.Y438H) in the LIG4 gene. The glutamine to arginine substitution on both alleles affects the catalytic domain of the gene, is contiguous to highly conserved ligase motifs, and is predicted to be damaging. Interestingly, this mutation maps close to the K424fs*20 mutation, which was previously reported in a patient with overt SCID and similar cellular phenotype, and other clinical features comparable to those described in our patient (microcephaly, growth retardation and cytopenia) [7]. Moreover, such phenotype has been unexpectedly associated with normal levels of Ig, as in our case. In particular, we detected an incomplete but severe block in precursor B cell differentiation and reduced numbers of B cells in peripheral blood along with a reduced number of CD4+ T-lymphocytes, which is presumably the result of a reduced efficiency of DSB repair affecting the ligation phase of V(D)J recombination, whose impact has been demonstrated to be different on various cell populations [7]. The essentially normal levels of serum Ig in the presence of severe primary B lymphopenia might indicate that a significant number of Ab-producing plasma cells could be generated from the few developing B cells. However, even in the presence of normal Ig levels, the IgG Ig receptor repertoire diversity is expected to be severely limited, accounting for aspects of the immunodeficiency [7]. Altogether, the genetic diagnosis of LIG4 deficiency, the history of recurrent infections and the available data on the patient’s immunological status pointed to a condition of immunodeficiency, whose degree, however, cannot be fully ascertained due to the unfortunate passing of the patient.

A second pathogenic mutation in LRIG2 (c.C2125T/p.R709X) was also detected, with the severe VUR and the related recurrent urinary tract infections and enuresis being the phenotypic counterpart of the UFS. To date only 5 patients with UFS2 have been reported (Table 2), and Stuart et al. [17] described this same p.R709X mutation in a homozygous state in two affected sisters with both facial and urinary phenotypes from a consanguineous Turkish family, and in heterozygosity in the unaffected parents.

**Table 2:** Overview of LRIG2 deficient patients (new and literature)

Patient^a^	Recessive mutations		Facial phenotype	Urinary tract phenotype	Constipation	Wilms tumour	Ref.
	Allele 1	Allele 2					
F 1.1	c.1230delA	c.1230delA	YES	YES	YES	NO	−17
F 1.2	c.1230delA	c.1230delA	YES	NO	NO	NO	−17
F 2	c.2088delC	c.1980–1981ins371	YES	YES	YES	NO	−17
F 3.1	c.2125C > T	c.2125C > T	YES	YES	YES	YES	−17
F3.2	c.2125C > T	c.2125C > T	YES	YES	Not known	NO	−17
NEW	c.2125C > T	c.2125C > T	NO	YES	NO	NO	This study
Hinman-Allen syndrome	c.1648C > T	c.2554A > T	NO	YES	NO	NO	−17

^a^Named as reported in single publications

The fact that our patient did not display the typical facial features, i.e., inverted smile giving an appearance of crying, demonstrates that for this given severe mutation facial features are not always present. A broad phenotypic variability has already been reported in families affected by UFS (OMIM#236730) sustained by mutations in Heparinase-2 gene (HPSE2), and it has been demonstrated that some affected individuals have only the facial or urinary tract phenotype [19]. The varied expression might be accounted for by modifier genes, epigenetics or environmental factors that remain to be identified.

Our findings confirm the hypothesis that many severe dysfunctional bladder problems might have an occult neurological background similar to UFS without expressing the grimace when smiling [20], and that LRIG2 might be implicated in the biological pathways underpinning the nonsyndromic VUR, which affects 1% of children and is commonly familial [17]. Stuart et al. described that, among 23 patients with Hinman-Allen syndrome (“non-neurogenic neurogenic bladder”), one case had urinary tract characteristics identical to those seen in UFS without typical facial features, and displayed compound heterozygous missense variants in LRIG2 [17].

In summary, we report in a single case how WGS can help piece together clinical puzzles, in which multiple causative mutations contribute to an overall complicated clinical phenotype. So far, few cases of LIG4 syndrome have been reported with detailed patients’ phenotypes, and the variety of these phenotypes is remarkable (Table 3), although in line with the consequences of hypomorphic mutations [21].

**Table 3:** Overview of LIG4 syndrome patients (new and literature)

Patient^a^	Recessive mutations		RS	Microcephaly	ID	Cytopenia	GR	DD	Malignancy	Other	Ref.
	Allele 1	Allele 2									
180BR	p.R278H	p.R278H	YES	NK	NO	NK	NK	NO	Leukemia		−5
3703	p.R814X	p.R814X	YES	YES	NK	YES	YES	YES	Leukemia		−8
X	p.M249V	p.K524 fs20X	YES	YES	Mild	YES	YES	YES	EBV- BCL		−6
2303	p.R580X	p.R814X	YES	YES	Mild	YES	YES	NK	MDS	Hypothiroidism, type 2 DM, hypogonadism	−9
99P0149	p.G469E	p.R814X	YES	YES	Mild	YES	YES	YES	NO	Atypical bone maturation	−9
Case 1	p.delK588	p.delK588	ND	YES	Mild	YES	YES	NO	NO		−10
Case 2	p.delK588	p.delK588	ND	YES	Mild	YES	YES	NO	NO		−10
10 Patients(9 families)	p.R814X	Variable truncating mutations	YES	YES	Mild	YES	YES	7 YES/ 3 NO^b^	NO	Skeletal abnormalities 6/10), primary ovarian failure (2/10), dysplastic kidney (2/10), anal atresia (1/10), corpus callosum dysgenesis (1/10)	−14
2304	p.R580X	p.R814X	YES	YES	Mild	NK	NO	NK	NO	Hypothiroidism, amenorrea	−9
411BR	p.R278H p.A3V, p.T9I	p.R278H p.A3V, p.T9I	YES	NO	Mild	YES	NO	YES	NO		−9
P1	p.H282L	p.K524 fs20X	YES	YES	SCID	YES	YES	NO	EBV- BCL		−7
Patient	S205L fsX29	K635R fsX10	YES	YES	SCID	YES	YES	YES	NO	Skeletal abnormalities, urethral valves, corpus callosum dysgenesis	−13
P-1	p.Q280R	p.K524 fs20X	YES	YES	SCID	NO	YES	NO	NO		−12
P-2	p.Q280R	p.K524 fs20X	YES	YES	SCID	NO	YES	NK	NO		−12
P2	p.H282L	p.K524 fs20X	YES	YES	SCID	NO	NO	YES	NO		−7
LIG4-1	p.delQ433	p.delQ433	YES	NO	SCID	YES	NO	NO	NO		−11
NEW	p.Y438H	p.Y438H	ND	YES	YES	YES	YES	YES	NO	Skeletal abnormalities, nail dystrophy	This study

*GR* Growth retardation, *ID* Immunodeficiency, *DD* Developmental delay, *RS* Radiosensitivity, *NK* Not known, *ND* Not determined, *EBV-BCL* EBV-associated B-cell lymphoma, *DM* Diabetes mellitus

^a^Named as reported in the single publications

^b^No association with the second mutation paired to p.R814X

Our findings open up the possibility that the clinical heterogeneity of some syndromes, such as LIG4 syndrome, could be a result of other homozygous mutations arising on a consanguineous genetic background. Thus, we believe that the discovery of additional LIG4 causative mutations from different families and the correlated phenotypes help to clarify the phenotype truly caused by LIG4 deficiency. Likewise, our report could help both in identifying the essential features of LRIG2 deficiency and in shedding light into the genetic background of dysfunctional voiding diseases, such as nonsyndromic VUR and Hinman-Allen syndrome.

Nevertheless, we are aware that the complexity of the WGS data obtained emphasizes the need of further research for comprehensive understanding of pathogenic mutations and concomitant genetic modifiers. Assigning a causal role for genes in disease states is one of the most challenging points in human genetic research and solicits a proactive collaboration between clinicians and researchers.

Finally, these results could have guided the clinical management of the child, if she had not passed away due to a fatal lung infection. The presence of a LIG4 mutation, accounting for impaired DNA damage repair mechanisms and, therefore, a higher incidence of malignancies and sensitivity to ionizing radiation and chemotherapy, would have been relevant both for prevention and early diagnosis of cancer. The family will certainly benefit from the disclosure of these results, in terms of prenatal or early diagnosis and treatment for both LIG4 syndrome and UFS.

## Additional file

Additional file 1: Table S1. Summary of variants across filtering steps. (DOCX 25 kb)

## Abbreviations

BW: Body weight
DSB: Double-strand break
HPSE2: Heparinase-2 gene
Ig: Immunoglobulins
LIG4: Ligase IV
LRIG2: Leucine-rich repeats and immunoglobulin-like domains 2 gene
NHEJ: Nonhomologous end-joining
SCID: Severe combined immunodeficiency
UFS: Urofacial syndrome
VUR: Vesicoureteral reflux
WGS: Whole Genome Sequencing
