# Supplementary material for: Two hits in one: whole genome sequencing unveils LIG4 syndrome and urofacial syndrome in a case report of a child with complex phenotype
Source: BMC Med Genet. 2016 Nov 17;17:84. doi: 10.1186/s12881-016-0346-7 (PMC5114772; doi:10.1186/s12881-016-0346-7)
Supplement: Additional file 1: Table S1. — Summary of variants across filtering steps. (DOCX 25 kb) [file 12881_2016_346_MOESM1_ESM.docx]

**Supplementary material**

Table S1: Summary of variants across filtering steps

| **Total variants** | 274858628 |
| --- | --- |
| **Indels+snps** | 4577191 |
| **Passed confidence criteria^1^** | 3429360 |
| **MAF < 0.1% in 1000G and ESP** | 239194 |
| **Homozygous** | 1606 |
| **Predicted deleterious^2^** | 12 |
| **Matching phenotype and recessive inheritance model^3^** | 2 |

**^1^** Confidence criteria: call quality > 20; read depth >20; passed by aligning algorithm

^2^ Frameshift, in-frame indel, or start/stop codon change; missense unless predicted tolerated by SIFT & PolyPhen-2; splice site loss up to 2 bases into intron or as predicted by MaxEntScan; Structural Variant

^3^ Immunodeficiency, hair abnormalities, clubbing of fingers and toes, growth abnormality, skeletal abnormalities; variant must be either absent from tested unaffected family members or present in heterozygous state
